# Exploring a Nuclear-Selective Radioisotope Delivery System for Efficient Targeted Alpha Therapy

**DOI:** 10.3390/ijms24119593

**Published:** 2023-05-31

**Authors:** Yuki Iizuka, Yoshiyuki Manabe, Kazuhiro Ooe, Atsushi Toyoshima, Xiaojie Yin, Hiromitsu Haba, Kazuya Kabayama, Koichi Fukase

**Affiliations:** 1 Department of Chemistry, Graduate School of Science, Osaka University, 1-1 Machikaneyama, Toyonaka 560-0043, Osaka, Japan; iizukay21@chem.sci.osaka-u.ac.jp (Y.I.); manabey12@chem.sci.osaka-u.ac.jp (Y.M.); 2 Forefront Research Center, Osaka University, 1-1 Machikaneyama, Toyonaka 560-0043, Osaka, Japan; 3 Division of Science, Institute for Radiation Sciences, Osaka University, 1-1 Machikaneyama, Toyonaka 560-0043, Osaka, Japan; toyo@irs.osaka-u.ac.jp; 4 Radioisotope Research Center, Institute for Radiation Sciences, Osaka University, 2-4 Yamadaoka, Suita 565-0871, Osaka, Japan; ooe@rirc.osaka-u.ac.jp; 5 RIKEN Nishina Center for Accelerator-Based Science, 2-1 Hirosawa, Wako 351-0198, Saitama, Japan; xiaojie.yin@riken.jp (X.Y.); haba@riken.jp (H.H.); 6 Center for Advanced Modalities and DDS, Osaka University, 1-1 Yamadaoka, Suita 565-0871, Osaka, Japan

**Keywords:** targeted alpha therapy (TAT), drug delivery system (DDS), radiolabeled antibody, α-ray, imaging, nuclear localization signal (NLS)

## Abstract

Targeted alpha therapy (TAT) has garnered significant interest as an innovative cancer therapy. Owing to their high energy and short range, achieving selective α-particle accumulation in target tumor cells is crucial for obtaining high potency without adverse effects. To meet this demand, we fabricated an innovative radiolabeled antibody, specifically designed to selectively deliver ^211^At (α-particle emitter) to the nuclei of cancer cells. The developed ^211^At-labeled antibody exhibited a superior effect compared to its conventional counterparts. This study paves the way for organelle-selective drug delivery.

## 1. Introduction

Drug delivery systems (DDSs) are a pivotal technology for achieving optimal drug efficacy and selectivity [1,2]. Alongside passive targeting using nanocarriers, including liposomes and polymers, active targeting, which involves the utilization of specific molecular interactions to achieve high specificity, has been widely used. Notably, antibodies are effective for achieving excellent specificity, as demonstrated by the numerous practical applications of antibody–drug conjugates (ADCs) [3,4]. Recently, higher-resolution drug delivery, that is, organelle-selective drug delivery, has garnered attention as a strategy for augmenting drug efficacy, based on its intensive accumulation at the target site [5]. In this study, we investigated a DDS designed to selectively target a specific organelle, specifically, the nuclei in target cells, and applied it in order to develop an efficient nuclear medicine.

Radiation therapy is a common cancer treatment modality, and in this regard, targeted radioisotope (RI) therapy has the advantage of being less burdensome than external beam radiation and can be applied to tumors that are difficult to irradiate externally, such as metastatic malignancies and brain tumors [6,7]. Several monoclonal antibodies armed with β-emitting radionuclides, including Zevalin (^90^Y-labeled rituximab) [8] and Bexxar (^131^I-labeled tositumomab) [9], have been developed as targeted RI medicines and have already been translated to practical use.

**Figure 1:**
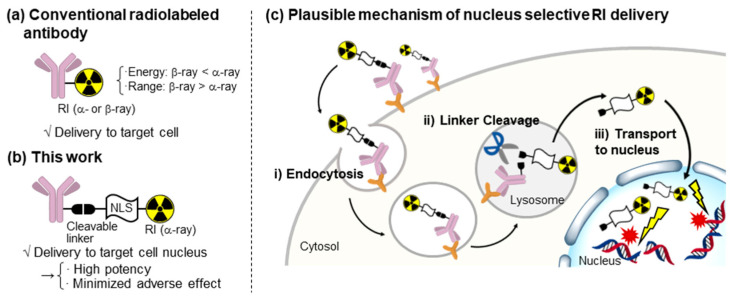
(**a**) Radiolabeled antibody and comparison between β- and α-rays. (**b**) Structure of radiolabeled antibody for delivering RI to the nuclei of target cells. (**c**) Plausible mechanism of nucleus-selective RI delivery.

Additionally, targeted alpha therapy (TAT) has garnered considerable interest in recent years [10,11]. Owing to the high energy and short range of α-rays, the selective accumulation of α-particles in target tumor cells can lead to remarkable therapeutic effects, with reduced adverse effects (Figure 1a). Additionally, α-rays primarily induce double-strand breaks (DSB), resulting in highly potent cytotoxicity. Therefore, TATs are being extensively investigated worldwide. For example, Xofigo, ^223^RaCl_2_ has been approved for practical use in the treatment of bone metastatic prostate cancer [12]. We are also actively engaged in TAT development with a focus on ^211^At as an α-particle source [13,14,15,16,17]. Particularly, Na^211^At, which leverages the halogen accumulation nature of the thyroid, is currently undergoing clinical trials for thyroid cancer therapy [13]. Furthermore, ^211^At-labeled α-methyl-L-tyrosine, which targets the cancer-associated amino acid transporter LAT1, exhibits remarkable antitumor activity [14]. Antibodies armed with α-particles also present a promising avenue for TAT. Since the pioneering study by Wilbur et al., several ^211^At-labeled antibodies have been reported [18,19], each demonstrating significant antitumor activity [20,21].

In this study, we developed a novel radiolabeled antibody that was designed to selectively deliver ^211^At to the nuclei of target cells for efficient TAT (Figure 1b). To accomplish this, we employed a nuclear localization signal (NLS) that acted as a tag for protein transport to the nucleus [22]. Specifically, the anti-cancer antibody was conjugated with NLS-functionalized ^211^At via a cleavable linker. This antibody conjugate was envisioned to behave as follows: (i) target cell recognition, followed by internalization via endocytosis; (ii) lysosomal cleavage of the linker to release an ^211^At-functionalized fragment; and (iii) accumulation of the released NLS-functionalized ^211^At in the nucleus of the target cell, and the subsequent induction of DNA damage (Figure 1c). Such high-resolution targeting of RIs was expected to result in improved selectivity and high efficacy, particularly in α-ray therapy, based on the high energy and short range of α-rays. The molecular design was validated using fluorescence imaging. Specifically, this imaging analysis underscored the importance of the membrane permeability of the payload with respect to lysosomal escape, which served as intermediates between steps (i) and (iii) stated above. Based on this discovery, we devised a design to facilitate this step by harnessing the dual function of decaborane ([B]_10_): as a carrier of ^211^At and a membrane permeabilizer. This is because [B]_10_ forms a stable complex with ^211^At [18,19], and is also a potent membrane permeabilizer owing to its chaotropic effect [23,24]. As expected, the developed nucleus-targeting ^211^At-labeled antibody showed superior efficacy. Therefore, in this study, we propose a high-resolution DDS with remarkable potency and selectivity as a novel drug development trend.

## 2. Results and Discussion

**Figure 2:**
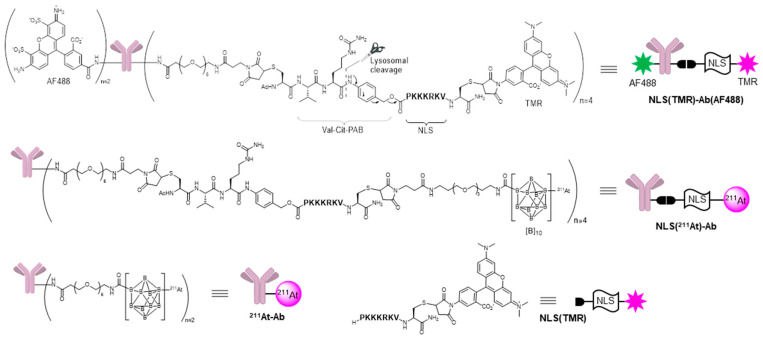
Structures of **NLS(TMR)-Ab(AF488)**, **NLS(^211^At)-Ab**, **^211^At-Ab**, and **NLS(TMR)**.

The molecules used in this study are shown in Figure 2. We used an anti-EpCAM antibody known for its selective binding to pancreatic cancer cells and cancer stem cells [25,26]. NLS(PKKKRKV)-functionalized TMR/^211^At was conjugated to the antibody via a valine–citrulline (Val-Cit) linker [27], which can be readily cleaved by lysosomal cathepsin. For the fluorescent probe, we designed and synthesized a doubly fluorescent-labeled antibody, **NLS(TMR)-Ab(AF488)**, in which an Alexa Fluor 488 (AF488)-labeled antibody was loaded with NLS-functionalized TMR (Scheme S1, Figure S1); the AF488 was used to track antibody dynamics, while the TMR served as an indicator of the intracellular dynamics of the payload. We also synthesized **NLS(^211^At)-Ab** as a radiolabeled antibody to deliver ^211^At into the nuclei of cancer cells (Scheme S2, Figure S2). These antibody conjugates were readily obtained via Fmoc solid-phase peptide synthesis (Fmoc SPPS) and maleimide-thiol ligation; after preparing the Val-Cit linker-conjugated NLS doubly functionalized with Cys by SPPS, the introduction of TMR or [B]_10_ at C-terminal Cys was followed by coupling with the antibody at the N-terminal Cys, yielding **NLS(TMR)-Ab(AF488)** or **NLS(^211^At)-Ab**, respectively. The TMR-labeled NLS (**NLS(TMR)**) was also prepared to trace the dynamics of the NLS-functionalized payload (Scheme S3), while **^211^At-Ab**, a conventional ^211^At-labeled antibody, was prepared as the control (Scheme S4, Figure S5). Notably, ^211^At was successfully introduced into [B]_10_, as reported by Wilbur et al. [18,19] during the preparation of both **NLS(^211^At)-Ab** and **^211^At-Ab** (Figures S3, S4, S6 and S7).

**Figure 3:**
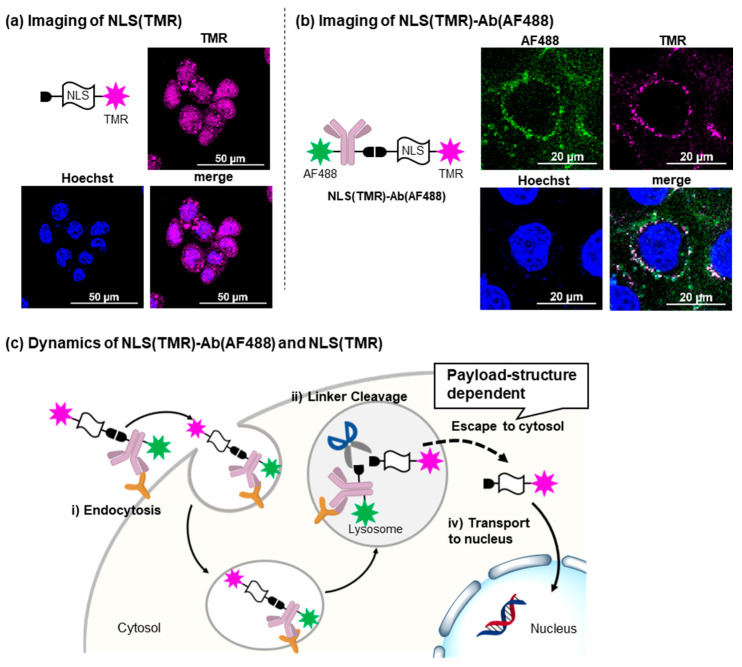
(**a**) Fluorescent imaging of **NLS(TMR)**. Cells were treated with **NLS(TMR)** via electroporation and images were obtained after incubation for 8 h. (**b**) Fluorescent imaging of **NLS(TMR)-Ab(AF488)**. The cells were treated with **NLS(TMR)-Ab(AF488)** (50 μg/mL) for 1 h and their nuclei were stained using Hoechst33342. (**c**) Dynamics of **NLS(TMR)-Ab(AF488)** and **NLS(TMR)**.

To verify the molecular design of our nucleus-selective DDS, live cell imaging was performed using PANC-1, the pancreatic cancer cell line. We first analyzed the intracellular dynamics of **NLS(TMR)** by introducing it to the cytosol via electroporation. **NLS(TMR)** was distributed throughout the cytosol and localized to the nucleus with a relatively high concentration, confirming the function of NLS in ensuring delivery to the nucleus (Figure 3a). Next, we analyzed the dynamics of **NLS(TMR)-Ab(AF488)** (Figure 3b, Figures S8 and S9). **NLS(TMR)-Ab(AF488)** was smoothly internalized into PANC-1 cells, and both AF488 and TMR fluorescence were observed in the cells. Importantly, after 1 h of incubation, the observed AF488 and TMR fluorescence partially unmerged, indicating that the Val-Cit linker was cleaved in lysosomes, allowing for the successful release of the payload. However, TMR fluorescence was observed as dots in the cells, suggesting that the NLS-functionalized TMR remained in the lysosomes and did not escape into the cytosol due to its low membrane permeability.

Overall, fluorescence imaging demonstrated the validity of the nucleus-selective DDS proposed in this study and its limitations (Figure 3c). The above imaging analysis confirmed the following three steps: (i) endocytosis into the target cells; (ii) lysosomal cleavage of the linker; and (iii) transport to the nucleus. However, TMR fluorescence was not observed when **NLS(TMR)-Ab(AF488)** was used, indicating that another critical step in the present method is lysosomal escape into the cytosol. Namely, the efficacy of the present nucleus-specific DDS depends on the physical properties (mainly the membrane permeability) of the payload. Based on these observations, we employed [B]_10_ as an ^211^At carrier, in consideration of its high membrane permeability due to its chaotropic effect [23,24].

**Figure 4:**
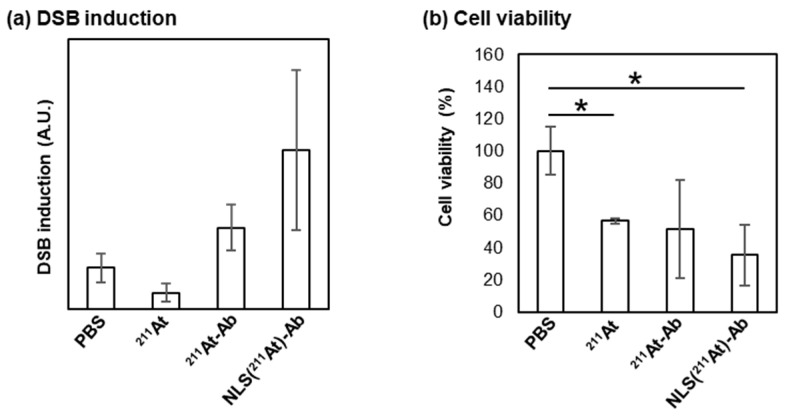
(**a**) DSB induction by **^211^At-Ab** and **NLS(^211^At)-Ab**. Cells were treated with each compound for 4 h. (**b**) Cell viability after **^211^At-Ab** or **NLS(^211^At)-Ab** treatment. The cells were treated with each compound for 1 h followed by incubation for 3.5 days. Data represents the results from three experiments (*n* = 3). The standard deviation (SD) is shown as the error bars. One-way ANOVA followed by Tukey’s test using GraphPad Prism 9: * *p* < 0.05.

The cytotoxicities of **NLS(^211^At)-Ab** and **^211^At-Ab** against PANC-1 cells were evaluated. After incubation for 4 h, **NLS(^211^At)-Ab** showed a greater capacity for inducing DSB than **^211^At-Ab** (Figure 4a and Figures S10–S13). Furthermore, the cell viability observed after the 4 day incubation period indicated that **NLS(^211^At)-Ab** exhibited a stronger cytotoxicity than **^211^At-Ab** (Figure 4b). These findings suggested that the accumulation of ^211^At resulted in potent cytotoxicity, thereby indicating the efficacy of the present nucleus-targeting strategy.

## 3. Materials and Methods

### 3.1. Synthesis of Compounds

The details of the synthetic procedure and the characterization data are shown in Supplementary Materials.

### 3.2. Fluorescent Imaging of NLS(TMR) Using Electroporation

PANC-1 cells were cultured using RPMI containing 10% FBS and 1% penicillin-streptomycin. PANC-1 cells were harvested by treating them with trypsin-EDTA solution, and cells in RPMI (1.5 × 10^6^ cells/mL, 390 mL) were transferred to 0.4 cm cuvettes. To the cuvette was added **NLS(TMR)** (6.03 mg) in RPMI (10 mL, final concentration: 10 mM), and the cells were exposed to the electric field (voltage: 200 V, capacitor: 900 mF). The cells were transferred to a 35 mm dish and incubated for 8 h at 37 °C. After washing with RPMI three times, the cells were treated with Hoechst33342 (10 μg/mL) in RPMI (100 mL) for 10 min at room temperature. After washing with RPMI three times, the cells were observed using confocal laser scanning microscopy (A1R, Nikon, Tokyo).

### 3.3. Fluorescent Imaging of NLS(TMR)-Ab(AF488)

PANC-1 cells were cultured using RPMI containing 10% FBS and 1% penicillin-streptomycin. PANC-1 cells were incubated for 2 days on a 35 mm glass-bottom dish. After suction of the medium, to this dish was added Hoechst33342 (10 μg/mL) in RPMI (100 mL), and the cells were incubated for 10 min at 37 °C. After washing with RPMI three times, to this dish was added **NLS(TMR)-Ab(AF488)** (PBS solution, 50 μg/mL) in RPMI (100 mL). After the cells were incubated for 1 h at 37 °C, the cells were observed using confocal laser scanning microscopy (A1R, Nikon, Tokyo, Japan).

### 3.4. Protocol for Evaluation of DSB Induction

PANC-1 cells (2 × 10^4^ cells/well, 96 well microplate) in RPMI (200 μL) were incubated for 1 day at 37 °C. After suctioning the medium, to the plate was added PBS or **^211^At-Ab** or **NLS(^211^At)-Ab** in PBS (100 mL, final concentration: 1 MBq/mL), and the cells were incubated for 4 h at 37 °C. After suctioning the medium, the cells were fixed with 4% PFA at room temperature for 30 min. After washing with PBS three times, the cells were treated with 0.1% Triton X-100 in PBS (100 mL) for 5 min. After washing with PBS three times, an AF488-labeled anti-gH2A.X antibody in PBS (100 mL, 2 mg/mL) was added, and the cells were incubated overnight at 4 °C. After washing with PBS three times, the cells were treated with Hoechst33342 in PBS (100 mL, 10 μg/mL) for 10 min at room temperature. After washing with PBS three times, the cells were observed using an All-in-One Fluorescence Microscope (KEYENCE CORPORATION, Osaka, Japan). The obtained images are shown in the Supplementary Materials (Figure S8).

The images were analyzed by Fiji (NIH). DSB induction was quantified as follows: the value of the total area stained with AF488-labeled anti-gH2A.X antibody over the value of the total area stained with Hoechst33342. Each parameter was set as follows: Brightness: 90–255 (Hoechst), 25–255 (AF488) for color threshold; size (micron^2): 0-infinity; and circularity: 0.00 for analyze particles. Three images were analyzed for all entries, and the mean and standard deviation were calculated.

### 3.5. Protocol for Evaluation of Cell Viability

PANC-1 cells (1 × 10^3^ cells/well, 96 well microplate) in RPMI (200 μL) were incubated for 1 day at 37 °C. After suctioning the medium, PBS, or **^211^At-Ab** or **NLS(^211^At)-Ab** in PBS (100 mL, final concentration: 1 MBq/mL) was added, and the cells were incubated for 3.5 h at 37 °C. After washing with PBS three times, RPMI containing 1% FBS (200 mL) was added, and the cells were incubated at 37 °C for 4 days. After incubation, to the plate was added Cell Counting Kit-8 solution (Dojindo, 20 mL, final concentration: 10%), and the cells were incubated for 3 h at 37 °C. The absorbance of formazan (450 nm) was measured by Infinite F50 (TECAN, Männedorf, Switzerland) in order to evaluate cell viability. The survival rate of each entry was standardized by calculating the survival rate of the untreated cells as 100%. Three trials were carried out for all entries, and the mean and standard deviation were calculated.

## 4. Conclusions

In summary, in order to develop an efficient TAT, a novel radiolabeled antibody was designed and synthesized to enable nucleus-selective RI transport, resulting in increased potency. This high-resolution drug delivery system was expected to be achieved by incorporating a signal peptide (NLS) and a cleavable Val-Cit linker, whose functions were confirmed via fluorescence imaging. The imaging analysis also highlighted the necessity for the payload to show membrane permeability in order to enable its escape from lysosomes. To overcome this challenge, we employed [B]_10_, which exhibited a dual function, as an ^211^At carrier and a membrane permeabilizer. To the best of our knowledge, this is the first report on the fabrication of an antibody conjugate oriented toward the organelle-selective delivery property of payloads. Organelle-selective drug delivery is a state-of-the-art drug delivery technology, and this study demonstrates its feasibility and clarifies design guidelines.

## Data Availability

Data are available in the article/Supplementary Material.

## Supplementary Materials

The supporting information can be downloaded at: https://www.mdpi.com/article/10.3390/ijms24119593/s1. References [28,29,30] are cited in the supplementary materials.
