# Supplementary material for: Exploring a Nuclear-Selective Radioisotope Delivery System for Efficient Targeted Alpha Therapy
Source: Int J Mol Sci. 2023 May 31;24(11):9593. doi: 10.3390/ijms24119593 (PMC10253442; doi:10.3390/ijms24119593)
Supplement: Supplementary file 1 [file ijms-24-09593-s001.zip › ijms-2391719-supplementary.pdf]

Supplementary information

Development of a Nuclear-selective Radioisotope Delivery System for Efficient Targeted Alpha Therapy

Yuki Iizuka, Yoshiyuki Manabe, Kazuhiro Ooe, Atsushi Toyoshima, Xiaojie Yin, Hiromitsu Haba, Kazuya Kabayama and Koichi Fukase

Table of Contents

1. General information 3

2. Synthesis procedures and characterization data 4

2-1. Synthesis of NLS(TMR)-Ab(AF488) 4

2-2. Synthesis of NLS(^{211}At)-Ab 8

2-3. Synthesis of NLS(TMR) 12

2-4. Synthesis of ^{211}At-Ab 15

3. Imaging analysis of NLS(TMR)-Ab(AF488) 18

3-1. Colocalization analysis 18

3-2. Imaging analysis of NLS(TMR)-Ab(AF488) after 4-h incubation 18

4. Results of DSB induction 19

4-1. Results of DSB induction (30 mins and 2 hours) 19

4-2. Images of DSB induction 19

5. Reference 21

1. General information

All reagents and solvents were obtained from commercial suppliers. They were used without further purification. All of the solvents used for the experiment were reagent grade or HPLC grade. Manual solid phase peptide synthesis (SPPS) was carried out in Reservoir-2 FRITS (Agilent Technologies). Automated solid phase peptide synthesis (SPPS) was carried out by Prelude® peptide synthesizer (Gyros protein technologies). Reversed-phase high-performance liquid chromatography (RP-HPLC) analysis was carried out by CLASS-VP system and LC solution system (SHIMADZU). The purity of synthesized peptide was evaluated by LC/MS with 1290 Infinity LC system (Agilent Technologies) connected to micrOTOF-QII (ESI-MS, Bruker) or prominence LC system (SHIMADZU) connected to compact (ESI-MS, Bruker). High-resolution mass spectra (HRMS) were obtained on an ESI-LTQ-Orbitrap XL (FTMS) mass spectrometer (Thermo Scientific). MALDI-TOF-MS spectra were obtained by Axima-CFR MALDI-TOF (SHIMADZU). The cell viability was measured by colorimetric NADP/NADPH Assay. The absorbance of formazan (450 nm) was measured by Infinite F50 (TECAN). The cell number was counted by EVE cell automatic cell counter (NanoEntek). For evaluation of DSB induction, All-in-One Fluorescence Microscope (KEYENCE, BZ-810) was used, and images were analyzed with Fiji (Wayne Rasband (NIH)). Microscopic images were taken on an inverted confocal microscope (A1R+, Nikon) with an LU-N4 laser unit. A 60× oil immersion objective lens (NA: 1.40) was used. The images were obtained with the Nikon software NIS-Elements. Gene Pulser® (Bio-Rad Laboratories, Inc.) was used for electroporation.

Materials and reagents:

PANC-1 cells were purchased from America Type Culture Collection (ATCC®, CRL-1469). Roswell Park Memorial Institute (RMPI-1640; 189-02025) and penicillin-streptomycin solution (×100; 168-23191) were purchased from Wako. Fetal bovine serum (FBS; 10270-106), Hoechst33342 (H3570), and trypan blue stain (0.4%; 15250-061) were purchased from Thermo Fisher Scientific. Cell Counting Kit-8 was purchased from Dojindo (343-07623). 96 well plate (3860-096) and 35 mm dish (3911-035) were purchased from IWAKI Brand Asahi glass CO., LTD. Anti-EpCAM antibody clone 1D12 was provided by Prof. Mashiko, Kinki University. Anti-gamma H2A.X (phosphor S139) antibody (ab11174) was purchased from abcam. 4% paraformaldehyde phosphate buffer solution was purchased from FUJIFILM Wako Chemicals (163-20145). Triton(R) X-100 was purchased from Nacalai Tesque (35501-15).

2. Synthesis procedures and characterization data

2-1. Synthesis of NLS(TMR)-Ab(AF488)

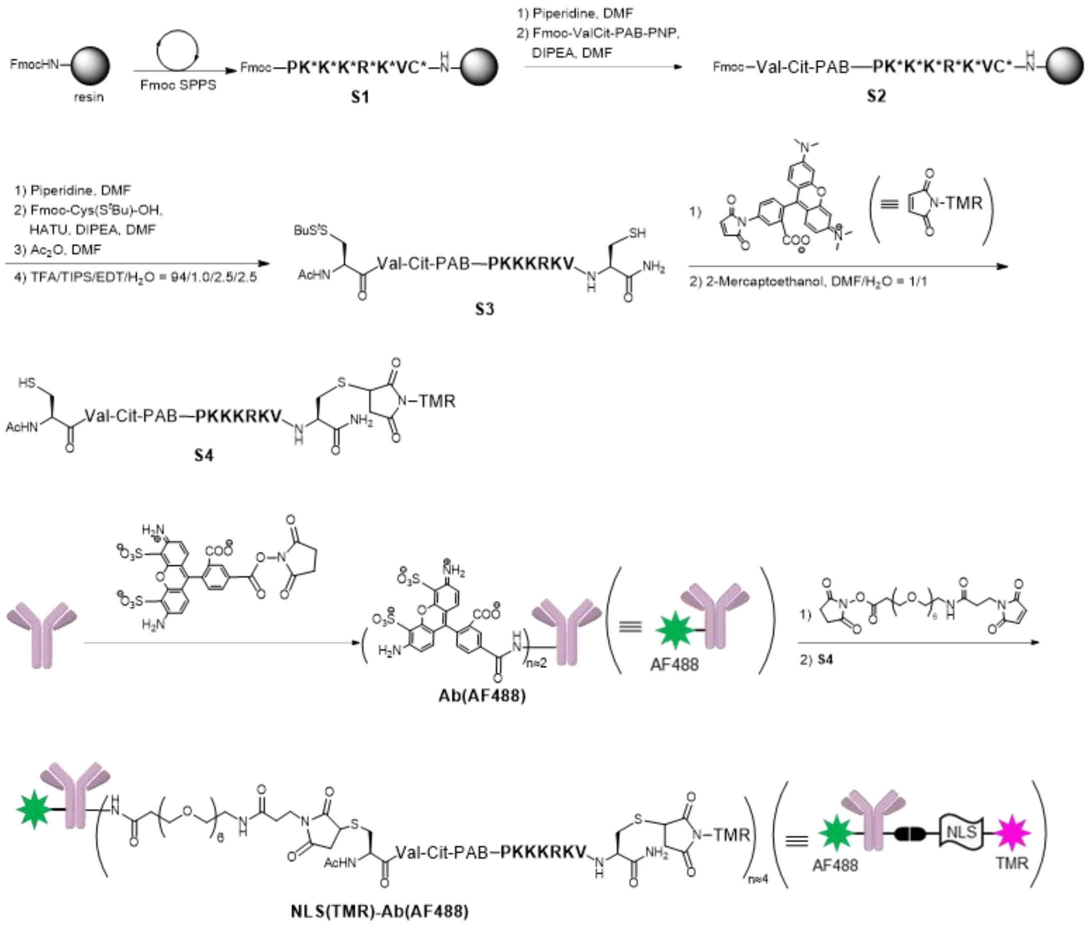

Scheme S1. Synthesis of **NLS(TMR)-Ab(AF488)**. Protected amino acids were indicated as AA*. K*: Lys(Boc); R*: Arg(Pbf); C*: Cys(Trt).

·Compound **S3**

Fmoc-Cys(Trt)-OH was assembled on Rink Amide resin (67.6 mg, 0.05 mmol, 0.74 mmol/g resin loading, novabiochem®) by manual Fmoc solid-phase peptide synthesis (SPPS) in 20 mL Agilent solid phase extraction (SPE) tube with adapter cap. Before the attachment of the first amino acid, the resin was swelled with DCM for 3 hours, and Fmoc deprotection was performed. To the swelled resin was added 20% piperidine in DMF (1 mL) at room temperature. After being shaken for 40 minutes at room temperature, the resin was washed with DMF (3 mL) five times. To the obtained resin was added the mixture of Fmoc-Cys(Trt)-OH (148 mg, 0.25 mmol, 5 eq), *N*-methyl morpholine (NMM, 55.0 µL, 0.50 mmol, 10 eq), HBTU (94.8 mg, 0.25 mmol, 5 eq), and HOBt (38.3 mg, 0.25 mmol, 5 eq) in DMF (1 mL) at room temperature. After being shaken vigorously under Ar gas for 30 minutes at room temperature, the resin was washed with DMF (3 mL) five times.

After loading cysteine to the resin, the subsequent building blocks were loaded by an automated peptide synthesizer (Prelude®) to obtain **S1**. Conditions for Fmoc deprotection: 20% piperidine in DMF (10 mL) for 4 minutes three times. Conditions for amino acid coupling: 120 mM Fmoc protected amino acids in DMF (2.5 mL, 6 eq.), 150 mM HCTU in DMF (4.0 mL, 12 eq.), and 75 mM NMM in DMF (4.0 mL, 6 eq.) for 30 minutes two times. Fmoc-Val-OH, Fmoc-Lys(Boc)-OH, Fmoc-Arg(Pbf)-OH were used. Half of the obtained resin was used for further reaction.

After construction of **S1**, the subsequent building blocks were loaded throughout manual SPPS in a 10 mL Agilent solid phase extraction (SPE) tube with an adapter cap. To the obtained **S1** was added 20% piperidine in DMF (1 mL) at room temperature. After being shaken for 40 minutes at room temperature, the resin was washed with DMF (3 mL) five times. To the obtained resin was added the mixture of Fmoc-ValCit-PAB-PNP (19.2 mg, 0.025 mmol, 1.0 eq.) and DIPEA (13.1 μL, 0.075 mmol, 3.0 eq.) in DMF (0.5 mL) at room temperature. After the reaction mixture was shaken for 1 hour at room temperature, the resin was washed with DMF (3 mL) five times. This coupling step was repeated, and the obtained resin was washed with DMF (3 mL) five times. To the obtained **S2** was added 20% piperidine in DMF (1 mL) at room temperature. After being shaken for 40 minutes at room temperature, the resin was washed with DMF (3 mL) five times. To the obtained resin was added the mixture of Fmoc-Cys(S^{t}Bu)-OH (54.0 mg, 0.125 mmol, 5 eq), NMM (27.5 μL, 0.25 mmol, 10 eq), HBTU (47.4 mg, 0.125 mmol, 5 eq) and HOBt (19.1 mg, 0.125 mmol, 5 eq) in DMF (0.5 mL) at room temperature. After being shaken for 1 hour at room temperature, the completion of coupling was confirmed by the Kaiser test. The resin was washed with DMF (3 mL) five times and DCM (3 mL) five times. To the obtained resin was added 20% piperidine in DMF (1 mL) at room temperature. After being shaken for 40 minutes at room temperature, the resin was washed with DMF (3 mL) five times. To the obtained resin was added 25% Ac_2O in DMF (0.5 mL) at room temperature. After being shaken for 5 minutes at room temperature, the resin was washed with DCM (3 mL) five times and dried *in vacuo*.

The dried resin was treated with a cocktail of TFA/TIPS/water/EDT (94/1.0/2.5/2.5) for 1 hour at room temperature. After filtration, the crude product was precipitated with ice-cold diethyl ether and collected. The crude precipitate was dried *in vacuo* and dissolved in methanol. The purification by RP-HPLC was carried out on a Nacalai Tesque 5C18-AR-300 column (10×250 mm) at a flow rate of 5 mL/min using a mobile phase of 0.1% TFA in water (Solvent A) and acetonitrile (Solvent B) (10 to 30% B gradient over 40 minutes, UV detection at 220 nm, 254 nm) to afford **S3** (5.18 mg, 13% yield) as a white solid.

HRMS (ESI-LTQ-Orbitrap XL, positive): *m/z* for $C_{71}H_{126}N_{22}O_{15}S_3$ $[M+2H]^{2+}$ calculated 812.4541, found 812.4549.

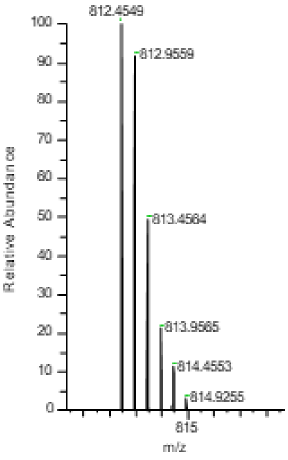

LC/MS *m/z* =1621: $[M+H]^+$, *m/z* =812: $[M+2H]^{2+}$; Analytical column (Nacalai Tesque 5C18-AR-300, 2.0×150 mm); 0.1% HCOOH in water (Solvent A) and acetonitrile (Solvent B) (2 to 98% B gradient over 48 minutes, 0.2 mL/min); UV detection at 220 nm.

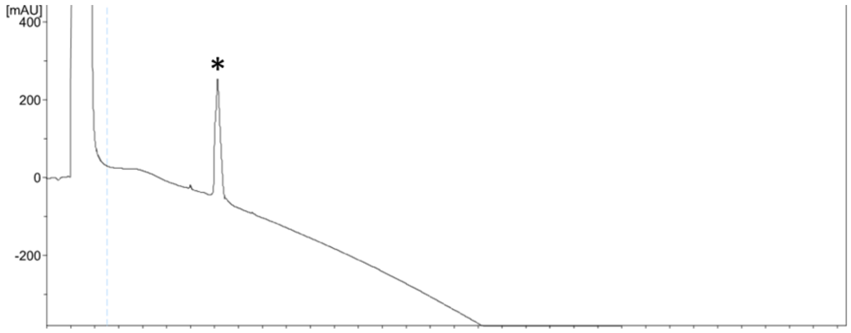

Tandem mass spectrometry (MS/MS), ESI-LTQ-Orbitrap XL, positive: Precursor ion = 812.5; CID power = 22.

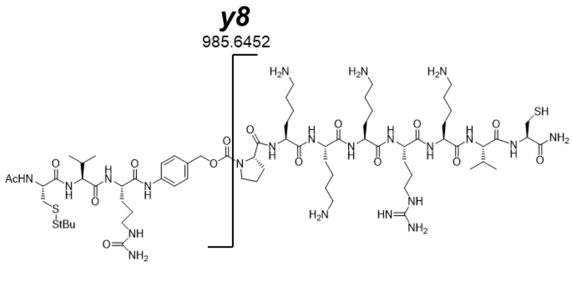

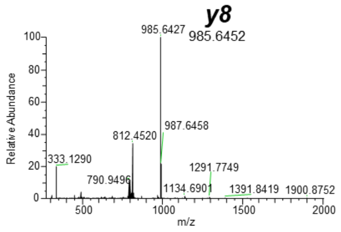

·Compound **S4**

To a solution of tetramethylrhodamine-5-maleimide (Invitrogen™, T6027) (0.25 mg, 0.51 μmol) in methanol (51.3 μL) was added **S3** (0.78 mg, 0.48 μmol) in DMF (44.7 μL) at room temperature. After incubation at 37 ºC for 2 hours, the reaction mixture was precipitated with ice-cold diethyl ether. The resultant precipitate was washed with ice-cold diethyl ether three times. To the resultant residue was added 20% 2-mercaptoethanol in DMF (93.8 μL) at room temperature. After being stirred at room temperature for 1 day, the reaction mixture was precipitated with ice-cold diethyl ether. The resultant precipitate was washed with ice-cold diethyl ether three times to give compound **S4**, which was used immediately for the next reaction without further purification.

·**NLS(TMR)-Ab(AF488)**

Anti-EpCAM antibody in PBS (400 μL) was transferred into a 30 kDa ultrafiltration tube (Amicon® Ultra-0.5mL Centrifugal Filters, Ultracel®-30K). The solution was centrifuged under 10,000 rpm for 10 minutes at 4 ºC. To the higher molecular weight fraction was added PBS (300 μL). The steps of centrifugation and PBS addition were repeated three times. Further conjugation reaction was carried out on the membrane filter. To the solution of antibody in PBS (90 μL) was added Alexa Fluor® 488 NHS Ester (6.40 μg, 0.001 μmol) in DMSO (10 μL, final concentration: 10 μM). After incubation for 20 minutes at room temperature, PBS (300 μL) was added, and the solution was centrifuged under 10,000 rpm for 10 minutes at 4 ºC. To the higher molecular weight fraction was added PBS (300 μL). The steps of centrifugation and PBS addition were repeated five times to give **Ab(AF488)**.

To the solution of **Ab(AF488)** in PBS (90 μL) was added 5 mM MAL-dPEG®$_6$-NHS ester in DMSO (10 μL, final concentration: 500 μM). After incubating for 30 minutes at 37 ºC, PBS (300 μL) was added and the solution was centrifuged under 10,000 rpm for 10 minutes at 4 ºC. To the higher molecular weight fraction was added PBS (300 μL). The steps of centrifugation and PBS addition were repeated five times. To the reaction solution (25 μL) was added **S4** (0.15 mg, 0.075 μmol) in DMSO (1.25 μL, final concentration: 3 mM). After incubating for 2 hours at 37 ºC, PBS (300 μL) was added, and the solution was centrifuged under 10,000 rpm for 10 minutes at 4 ºC. To the higher molecular weight fraction was added PBS (300 μL). The steps of centrifugation and PBS addition were repeated three times. The following steps are carried out for the capping of unreacted maleimide. To the solution in PBS (90 μL) was added cysteine (58.6 μg, 0.10 μmol) in PBS (10 μL, final concentration: 1 mM). After incubating for 30 minutes at 37 ºC, PBS (300 μL) was added, and the suspension was centrifuged under 10,000 rpm for 10 minutes at 4 ºC. To the higher molecular weight fraction was added PBS (300 μL). The steps of centrifugation and PBS addition were repeated five times to give **NLS(AF488)-Ab(TMR)**.

<Results of MALDI-MS analysis>

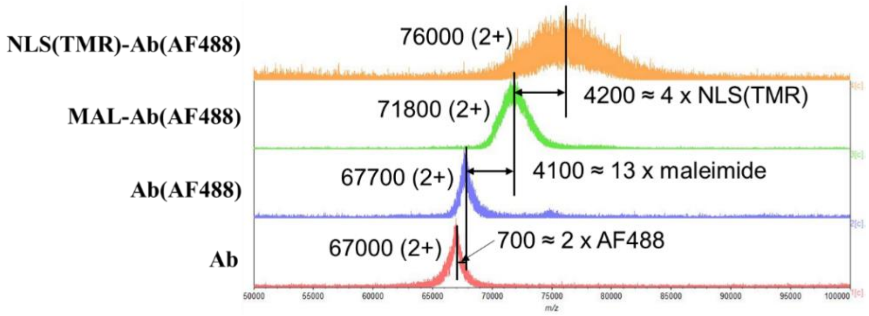

Figure S1. MALDI-MS analysis. Red: anti-EpCAM antibody (**Ab**); blue: **Ab(AF488)**; green: maleimide- and AF488-labeled antibody (**MAL-Ab(AF488)**); orange: **NLS(TMR)-Ab(AF488)**. The average loading ratio of AF488 and TMR was estimated to be 2 and 4, respectively.

2-2. Synthesis of NLS(^{211}At)-Ab

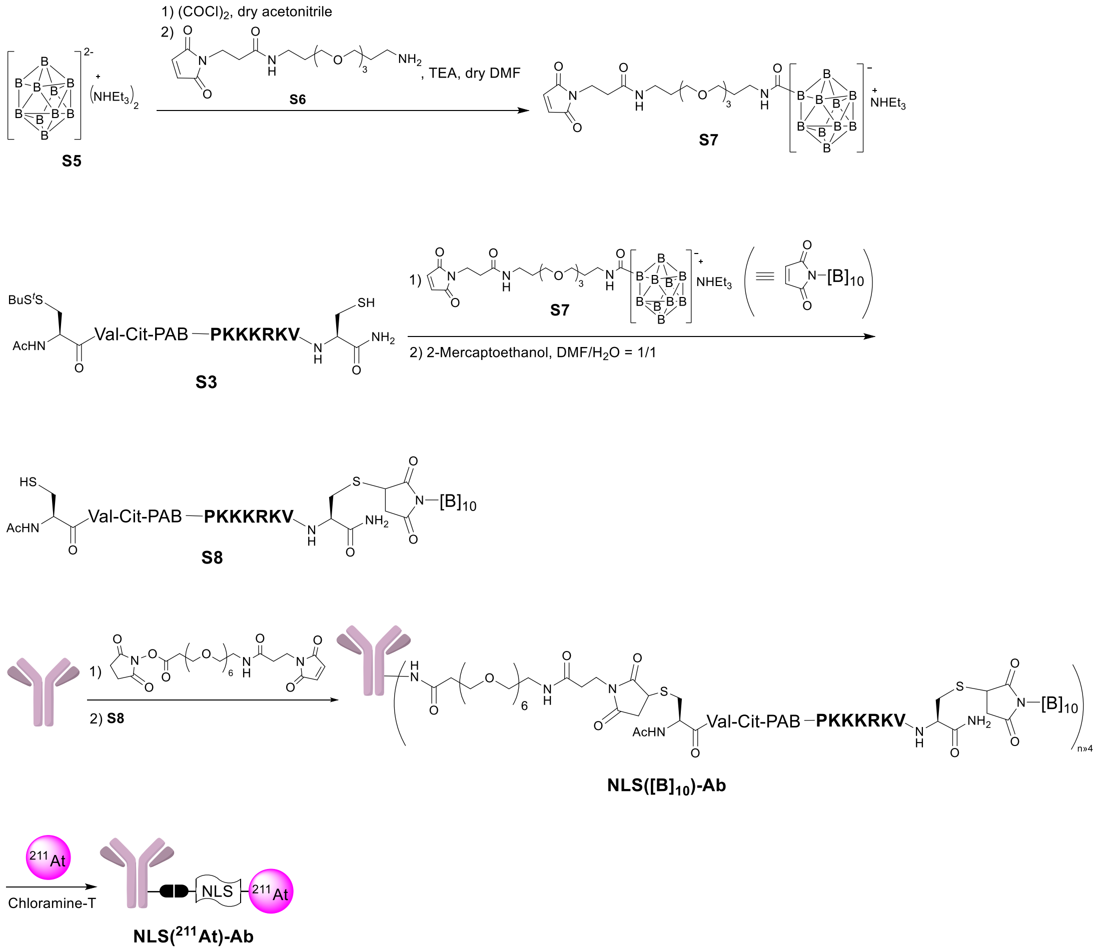

Scheme S2. Synthesis of **NLS(^{211}At)-Ab**

·Compound **S7**

To a solution of **S5**[1] (4.97 mg, 15.3 μmol) in dry acetonitrile (100 μL) was added oxalyl chloride (2.69 μL, 30.6 μmol) at room temperature. After being stirred at room temperature for 1 hour, the reaction mixture was concentrated *in vacuo*. To the resultant residue were added **S6**[2] (3.80 mg, 10.2 μmol) in dry DMF (200 μL) and triethylamine (4.26 μL, 30.6 μmol) at room temperature. After being stirred at room temperature for 3 hours, the reaction mixture was concentrated *in vacuo*. The residue was purified by silica gel column chromatography (6 to 50% methanol in 1% TEA in chloroform) to give compound **S7** (2.47 mg, 3.40 μmol, 33%) as a colorless oil. Similar to the report by Wilbur *et al.*,[3] we were unsuccessful in obtaining any mass spectral data for the decaborane derivative **S7**.

·Compound **S8**

To a solution of **S3** (1.00 mg, 0.62 μmol) in methanol (60 μL) were added **S7** (0.45 mg, 0.62 μmol) in methanol (21 μL) and 1% TEA in methanol (43 μL) at room temperature. After being stirred at 37 ºC for 1 hour, the reaction mixture was precipitated with ice-cold diethyl ether. The resultant precipitate was washed with ice-cold diethyl ether three times. To the resultant residue (0.18 mg, 0.075 μmol) was added 20% 2-mercaptoethanol in DMF/water = 1/1 (93.8 μL) at room temperature. After being stirred at room temperature for 1 day, the reaction mixture was precipitated with ice-cold diethyl ether. The resultant precipitate was washed with ice-cold diethyl ether three times to give compound **S8**, which was used immediately for the next reaction without further purification.

·NLS($[B]_{10}$)-Ab

Anti-EpCAM antibody in PBS (400 μL) was transferred into a 30 KDa ultrafiltration tube (Amicon® Ultra-0.5mL Centrifugal Filters, Ultracel®-30K). The solution was centrifuged under 10,000 rpm for 10 minutes at 4 ºC. To the higher molecular weight fraction was added PBS (300 μL). The steps of centrifugation and PBS addition were repeated three times. Further conjugation reaction was carried out on the membrane filter. To the solution of antibody in PBS (90 μL) was added 5 mM MAL-dPEG®$_6$-NHS ester in DMSO (10 μL, final concentration: 500 μM). After incubating for 30 minutes at 37 ºC, PBS (300 μL) was added, and the solution was centrifuged under 10,000 rpm for 10 minutes at 4 ºC. To the higher molecular weight fraction was added PBS (300 μL). The steps of centrifugation and PBS addition were repeated five times. To the reaction solution in PBS (90 μL) was added **S8** (0.67 mg, 0.30 μmol) in DMSO (10 μL, 30 mM, final concentration: 3 mM) at room temperature. After incubation for 2 h at 37 ºC, PBS (300 μL) was added, and the solution was centrifuged under 10,000 rpm for 10 minutes at 4 ºC. To the higher molecular weight fraction was added PBS (300 μL). The steps of centrifugation and PBS addition were repeated three times. The following steps were carried out for the capping of unreacted maleimide. To the solution in PBS (90 μL) was added cysteine (58.6 μg, 0.10 μmol) in PBS (10 μL, 10 mM, final concentration: 1 mM). After incubating for 30

minutes at 37 ºC, PBS (300 μL) was added, and the solution was centrifuged under 10,000 rpm for 10 minutes at 4 ºC. To the higher molecular weight fraction was added PBS (300 μL). The steps of centrifugation and PBS addition were repeated five times to give **NLS([B]$_{10}$)-Ab**.

<Results of MALDI-MS analysis>

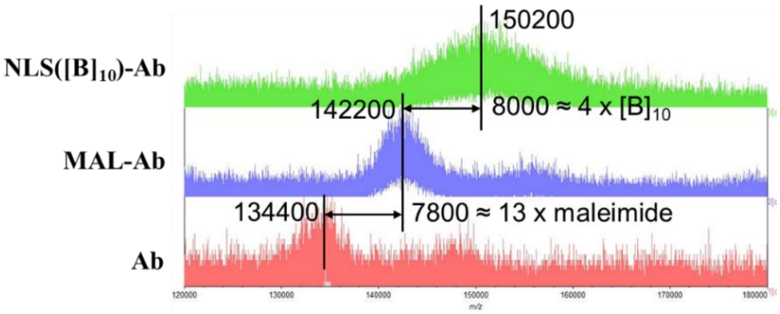

Figure S2. MALDI-MS analysis. Red: anti-EpCAM antibody (**Ab**); blue: maleimide-labeled antibody (**MAL-Ab**); green: **NLS([B]$_{10}$)-Ab**. The average loading ratio of compound **S8** was estimated to be 4.

·**NLS(^{211}At)-Ab**

To a solution of chloramine-T in distilled water (0.5 mg/mL) were added Na[^{211}At] solution in water (5.6 MBq/9.8 μL) and PBS (21.7 μL). To this solution was added **NLS([B]$_{10}$)-Ab** (10 μg) in PBS (62.5 μL) at room temperature. After incubation for 10 minutes at room temperature, the reaction was quenched with $Na_2S_2O_5$ (0.5 mg/mL in distilled water, 6 μL). The solution was transferred to Cosmopin filter H (GE Healthcare, 17-0045-02) filled with Sephadex G-25 DNA grade (Nacalai Tesque, 06540-34). The solution was centrifuged under 3,400 rpm for 2 minutes at room temperature to give **NLS(^{211}At)-Ab**. The radiation amount was measured by a Ge semiconductor detector (CANBERRA, BE2020). The radiochemical yield of **NLS(^{211}At)-Ab** was calculated to 33%. Purity of **NLS(^{211}At)-Ab** was checked by TLC (eluent: methanol) using a radio imager as a detector.

<Results of measurement by Ge semiconductor detector>

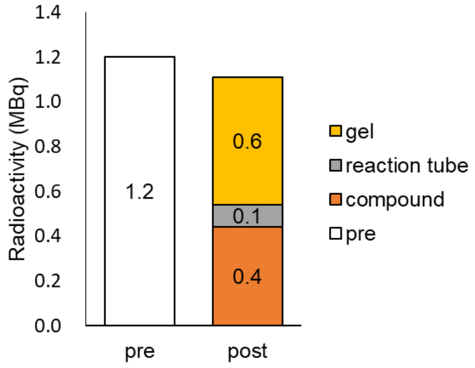

Figure S3. Result of gamma-ray measurement of **NLS(^{211}At)-Ab**. Pre: radioactivity of reaction mixture considering decay of ^{211}At. Post: each radiation amount after purification (gel: absorption to filter and gel used for purification; reaction tube: absorption to reaction tube; compound: **NLS(^{211}At)-Ab**.

<Results of radio TLC>

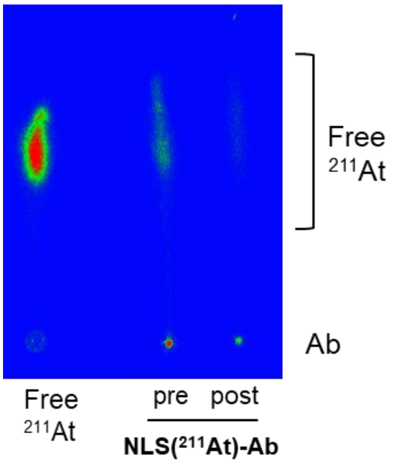

Figure S4. Radio TLC of **NLS(^{211}At)-Ab**. Eluent: methanol. Detection: radio imager. Pre: before purification. Post: after purification.

2-3. Synthesis of NLS(TMR)

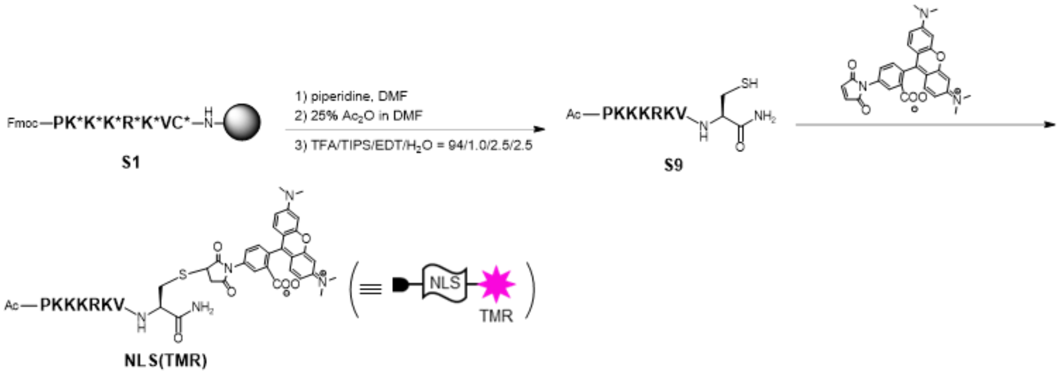

Scheme S3. Synthesis of **NLS(TMR)**. Protected amino acids were indicated as AA*. K*: Lys(Boc); R*: Arg(Pbf); C*: Cys(Trt).

·Compound **S9**

To **S1** (6.25 μmol) was added 20% piperidine in DMF (125 μL) at room temperature. After being shaken for 40 minutes at room temperature, the resin was washed with DMF five times. To the obtained resin was added 25% Ac_2O in DMF (125 μL) at room temperature. After being shaken for 5 minutes at room temperature, the resin was washed with DMF (125 μL) five times and with DCM (125 μL) five times, and then dried *in vacuo*.

The dried resin was treated with a cocktail of TFA/TIPS/water/EDT (94/1.0/2.5/2.5) for 1 hour at room temperature. After filtration, the crude product was precipitated with ice-cold diethyl ether and collected. The crude precipitate was dried *in vacuo* and dissolved in methanol. The purification by RP-HPLC was carried out on a Nacalai Tesque 5C18-AR-300 column (4.6×250 mm) at a flow rate of 1 mL/min using a mobile phase of 0.1% TFA in water (Solvent A) and acetonitrile (Solvent B) (2 to 15% B gradient over 13 minutes, UV detection at 220 nm, 254 nm) to afford **S9** (6.42 mg, 58% yield) as a white solid.

HRMS (ESI-LTQ-Orbitrap XL, positive): *m/z* for $C_{45}H_{86}N_{16}O_9S$ $[M+3H]^{3+}$ calculated 343.2234, found 343.2233, $[M+2H]^{2+}$ calculated 514.3315, found 514.3317, $[M+H]^+$ calculated 1027.6558, found 1027.6559.

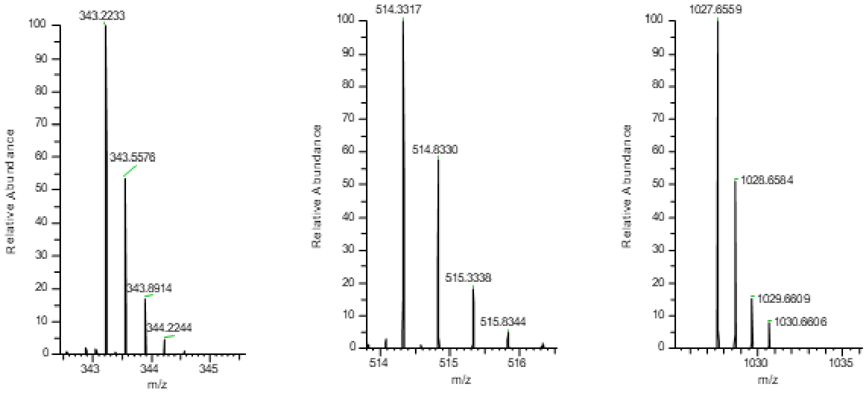

LC/MS HPLC conditions: analytical column (Nacalai Tesque 5C18-AR-300, 2.0×150 mm); 0.1% HCOOH in water (Solvent A) and acetonitrile (Solvent B) (2 to 15% B gradient over 13 minutes, 0.2 mL/min); UV detection at 220 nm, Detected MS: *m/z* =1027: $[M+H]^+$, *m/z* =514: $[M+2H]^{2+}$;

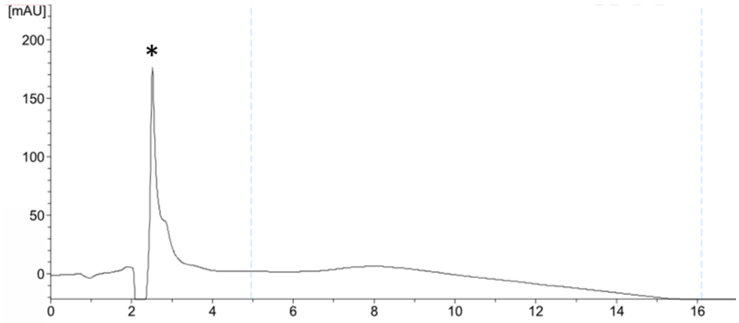

Tandem mass spectrometry (MS/MS), ESI-LTQ-Orbitrap XL, positive: Precursor ion = 343.2; CID power = 27.

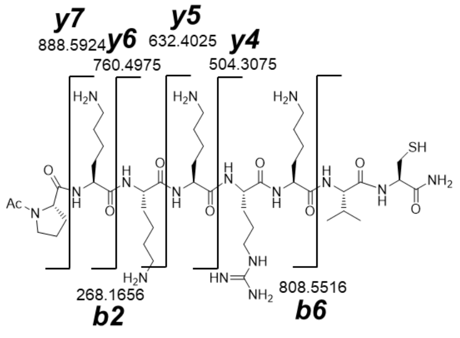

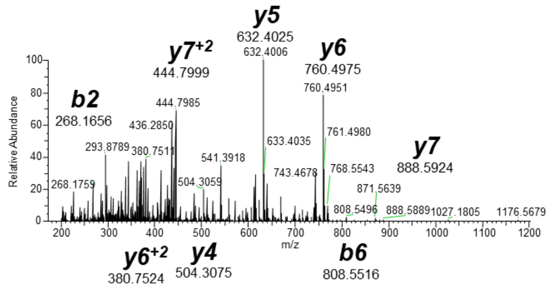

·**NLS(TMR)**

To tetramethylrhodamine-5-maleimide (Invitrogen™, T6027, 0.20 mg, 0.42 μmol) was added a solution of **S9** (0.36 mg, 0.35 μmol) in DMF (70 μL) at room temperature. After being incubated at 37 ºC for 2 hours, the reaction mixture was precipitated with ice-cold diethyl ether. The resultant precipitate was washed with ice-cold diethyl ether three times to give compound **NLS(TMR)**.

HRMS (ESI-LTQ-Orbitrap XL, positive): *m/z* for $C_{45}H_{86}N_{16}O_9S$ $[M+4H]^{4+}$ calculated 377.9603, found 377.9607, $[M+3H]^{3+}$ calculated 503.6114, found 503.6116, $[M+2H]^{2+}$ calculated 754.9134, found 754.9139, $[M+H]^{+}$ calculated 1508.8195, found 1508.8214.

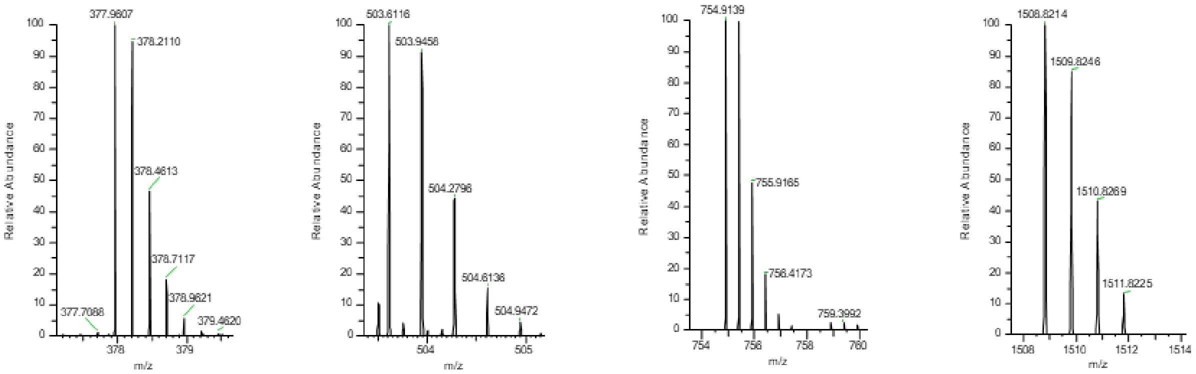

LC/MS *m/z* =1509: $[M+H]^{+}$, *m/z* =754: $[M+2H]^{2+}$; Analytical column (Nacalai Tesque 5C18-AR300, 2.0×150 mm); 0.1%HCOOH in water (Solvent A) and acetonitrile (Solvent B) (2 to 98% B gradient over 48 min, 0.2mL/min; UV detection at 220 nm)

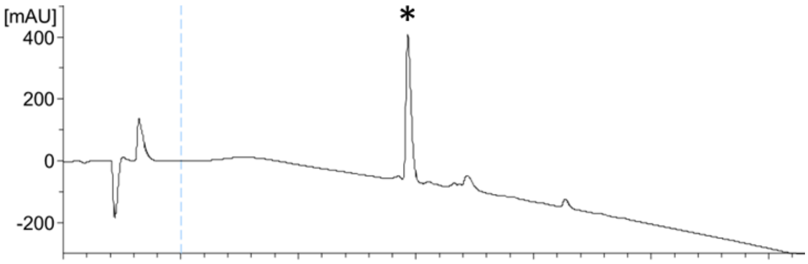

Tandem mass spectrometry (MS/MS), ESI-LTQ-Orbitrap XL, positive: Precursor ion = 378; CID power = 25.

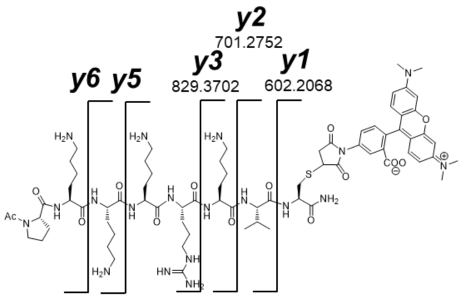

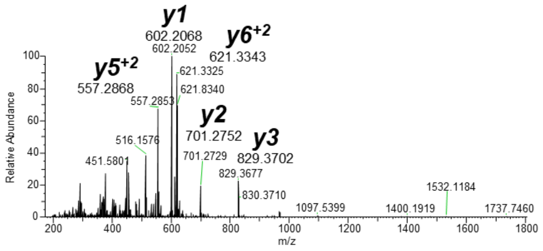

2-4. Synthesis of ^{211}At-Ab

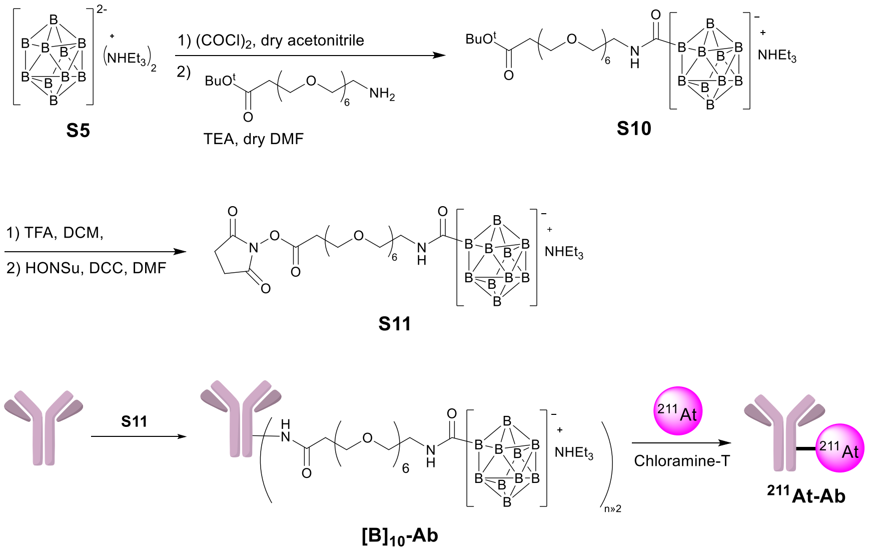

Scheme S4. Synthesis of ^{211}**At-Ab**

·Compound **S10**

To the solution of compound **S5** (56.5 mg, 0.17 mmol) in dry acetonitrile (1.16 mL) was added oxalyl chloride (30.6 μL, 0.35 mmol) at room temperature. After being stirred at room temperature for 2 hours, the reaction mixture was concentrated *in vacuo*. To a solution of the resultant residue in dry DMF (1.16 mL) were added ^{t}BuOOC-PEG6-NH_2 (47.5 mg, 0.12 mmol) and TEA (48.5 μL, 0.35 mmol) at room temperature. After being stirred at room temperature for 1 hour, the reaction mixture

was concentrated *in vacuo*. The residue was purified by silica gel column chromatography (6 to 50% methanol in chloroform) to give compound **S10** (78.2 mg, 89%) as a colorless oil. Similar to the report by Wilbur *et al.*,[3] we were unsuccessful in obtaining any mass spectral data for the decaborane derivative **S10**.

·Compound **S11**

To **S10** (5.10 mg, 6.70 μmol) was added TFA/dry dichloromethane = 1/1 (0.5 mL) at room temperature. After being stirred at room temperature for 90 minutes, the reaction mixture was concentrated *in vacuo*. To a solution of the resultant residue in dry DMF (0.2 mL) were added *N*-hydroxysuccinimide (0.92 mg, 8.04 μmol) and DCC (2.07 mg, 10.1 μmol) at room temperature. After being stirred at room temperature for 1 hour, the reaction mixture was concentrated *in vacuo* to give compound **S11**, which was used immediately for the next reaction without further purification. Similar to the report by Wilbur *et al.*,[3] we were unsuccessful in obtaining any mass spectral data for the decaborane derivative **S11**.

·**[B]$_{10}$-Ab**

Anti-EpCAM antibody in PBS (400 μL) was transferred into a 10 KDa ultrafiltration tube (Amicon® Ultra-0.5mL Centrifugal Filters, Ultracel®-10K). The solution was centrifuged under 10,000 rpm for 10 minutes at 4 ºC. To the higher molecular weight fraction was added PBS (300 μL). The steps of centrifugation and PBS addition were repeated three times. Further conjugation reaction was carried out on the membrane filter. To the solution of antibody in PBS (95 μL) was added **S11** (60.2 μg, 0.075 μmol) in DMSO (5 μL, 15 mM, final concentration: 0.75 mM). After incubating for 15 minutes at 37 ºC, PBS (300 μL) was added, and the solution was centrifuged under 10,000 rpm for 10 minutes at 4 ºC. To the higher molecular weight fraction was added PBS (300 μL). The steps of centrifugation and PBS addition were repeated five times to give **[B]$_{10}$-Ab**.

<Results of MALDI-MS analysis>

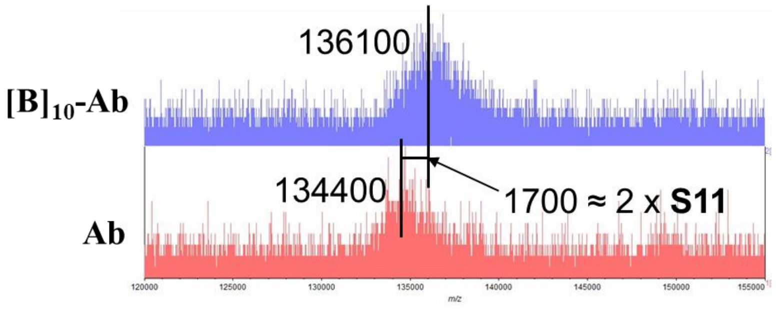

Figure S5. MALDI-MS analysis. Red: anti-EpCAM antibody (**Ab**); blue: **[B]$_{10}$-Ab**. The average loading ratio of compound **S11** was estimated to be 2.

·^{211}At-Ab

To a solution of chloramine-T in distilled water (0.5 mg/mL) were added Na[^{211}At] solution in water (5.6 MBq/9.8 μL) and PBS (21.7 μL). To this solution was added **[B]$_{10}$-Ab** (10 μg) in PBS (62.5 μL) at room temperature. After incubation for 10 minutes at room temperature, the reaction was quenched with $Na_2S_2O_5$ (0.5 mg/mL in distilled water, 6 μL). The solution was transferred to Cosmopin filter H (GE Healthcare, 17-0045-02) filled with Sephadex G-25 DNA grade (Nacalai Tesque, 06540-34). The solution was centrifuged under 3,400 rpm for 2 minutes at room temperature to give **^{211}At-Ab**. The radiation amount was measured by a Ge semiconductor detector (CANBERRA, BE2020). The radiochemical yield of **^{211}At-Ab** was calculated to 42%. Purity of **^{211}At-Ab** was checked by TLC (eluent: methanol) using a radio imager as a detector.

<Results of measurement by Ge semiconductor detector>

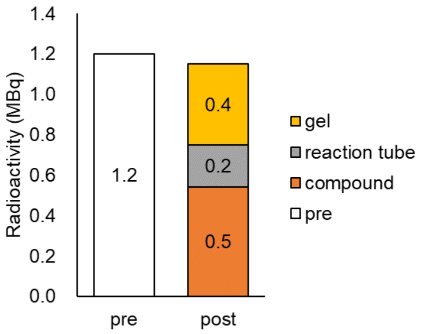

Figure S6. Result of gamma-ray measurement of **^{211}At-Ab**. Pre: radioactivity of reaction mixture considering decay of ^{211}At. Post: each radiation amount after purification (gel: absorption to filter and gel used for purification; reaction tube: absorption to reaction tube; compound: **^{211}At-Ab**).

<Results of radio TLC>

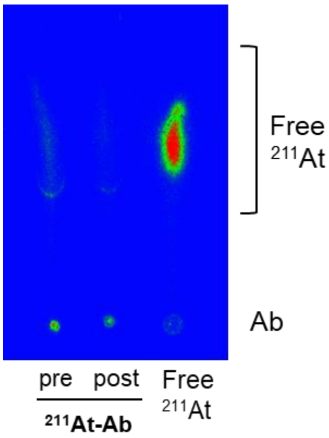

Figure S7. TLC of **^{211}At-Ab**. Eluent: methanol. Detection: radio imager. Pre: before purification. Post: after purification.

3. Imaging analysis of NLS(TMR)-Ab(AF488)

3-1. Colocalization analysis

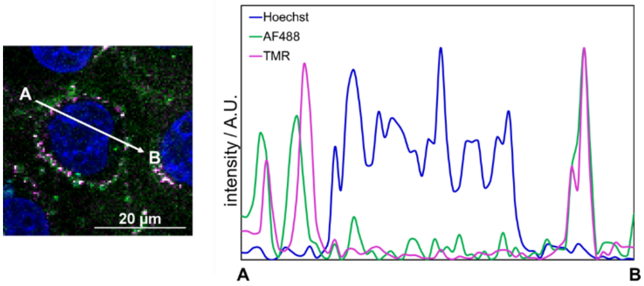

Figure S8. Colocalization analysis of imaging data shown in Figure 3b.

3-2. Imaging analysis of NLS(TMR)-Ab(AF488) after 4-h incubation

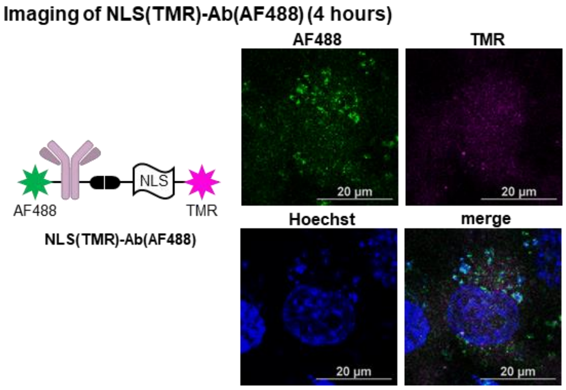

Figure S9. Fluorescent imaging of **NLS(TMR)-Ab(AF488)**. The cells were treated with **NLS(TMR)-Ab(AF488)** (50 µg/mL) for 4 hour and their nuclei was stained using Hoechst33342.

4. Results of DSB induction

4-1. Results of DSB induction (30 mins and 2 hours)

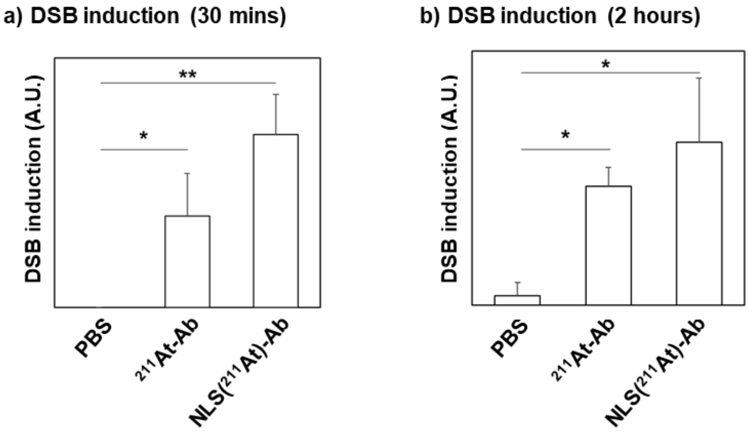

Figure S10. DSB induction by **^{211}At-Ab** and **NLS(^{211}At)-Ab**. a) Cells were treated with each compound for 30 mins. b) Cells were treated with each compound for 2 hours. Data represent the results from three experiments (n = 3). The standard deviation (SD) is shown as the error bars. One-way ANOVA followed by Tukey's test using GraphPad Prism 9: *p<0.05; **p<0.01

4-2. Images of DSB induction

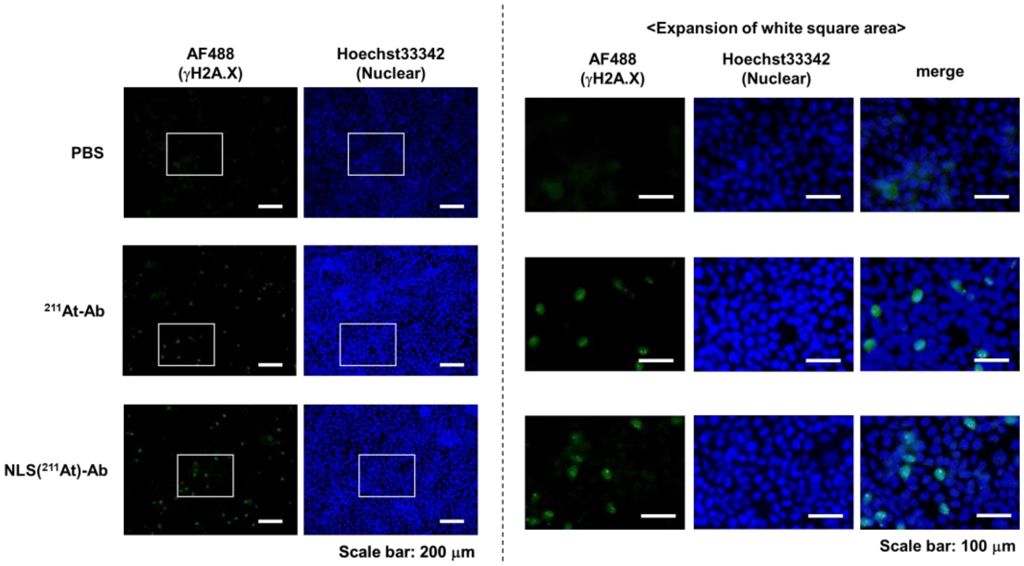

Figure S11. Representative data of DSB induction imaging after 30 min-incubation. γH2A.X was stained with AF488-labeled anti-γH2A.X antibody and cell nuclei were stained with Hoechst33342. Right: expansion of white square area.

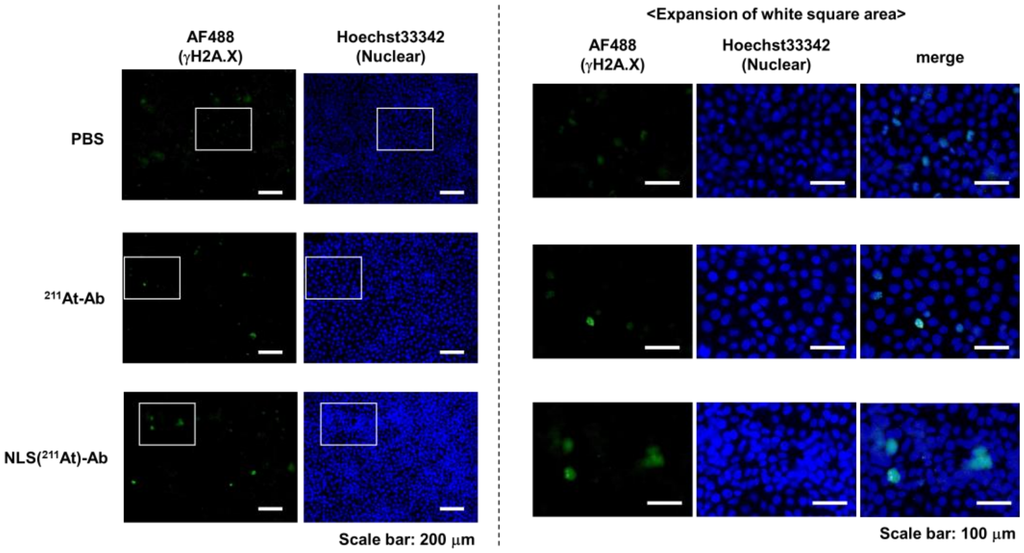

Figure S12. Representative data of DSB induction imaging after 2 h-incubation. γH2A.X was stained with AF488-labeled anti-γH2A.X antibody and cell nuclei were stained with Hoechst33342. Right: expansion of white square area.

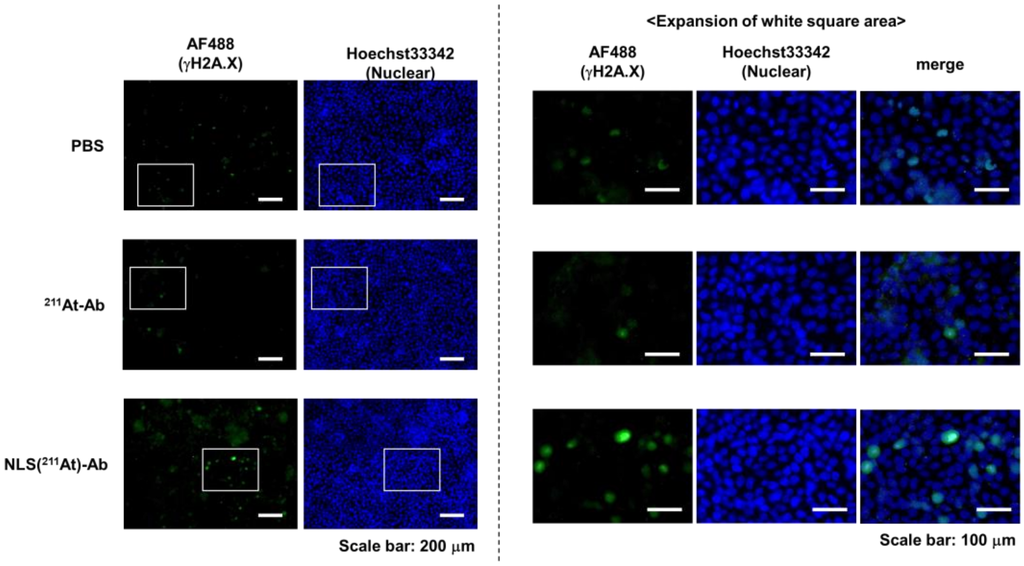

Figure S13. Representative data of DSB induction imaging after 4 h-incubation. γH2A.X was stained with AF488-labeled anti-γH2A.X antibody and cell nuclei were stained with Hoechst33342. Right: expansion of white square area.

5. Reference

1. M. F. Hawthorne, R. L. Pilling and W. H. Knoth, in *Inorg. Synth.*, 1967, pp. 16-19.
2. G. Zheng, L. Cochella, J. Liu, O. Hobert and W.-h. Li, *ACS Chem. Biol.*, 2011, **6**, 1332-1338.
3. D. S. Wilbur, M.-K. Chyan, D. K. Hamlin and M. A. Perry, *Bioconjugate Chem.*, 2009, **20**, 591-602.
